# Heparin-Induced Thrombocytopenia Following Tinzaparin Administration: A Case Report

**DOI:** 10.7759/cureus.93248

**Published:** 2025-09-26

**Authors:** Laila Khallaf, Praveenkumar Katarki, Nawaid Ahmad, Thimmegowda Govindagowda

**Affiliations:** 1 Internal Medicine, Shrewsbury and Telford Hospital NHS Trust, Telford, GBR; 2 Acute Medicine, Shrewsbury and Telford Hospital NHS Trust, Telford, GBR; 3 Respiratory and Acute Medicine, Shrewsbury and Telford Hospital NHS Trust, Telford, GBR

**Keywords:** 4ts score, anticoagulation, endovascular aneurysm repair, fondaparinux, heparin-induced thrombocytopenia, syncope, thrombocytopenia, tinzaparin

## Abstract

Heparin-induced thrombocytopenia (HIT), a severe immune-mediated reaction, presents a significant diagnostic challenge, particularly with atypical symptoms like syncope or when induced by low-molecular-weight heparin (LMWH). This report describes the case of a 77-year-old male who developed severe thrombocytopenia following tinzaparin administration for bridging therapy after an elective endovascular aneurysm repair (EVAR). The patient presented to the emergency department with syncope, an uncommon manifestation of HIT. Initial investigations revealed isolated thrombocytopenia, and a high clinical suspicion based on a 4Ts score of 6 prompted the immediate discontinuation of tinzaparin and initiation of fondaparinux. The diagnosis was subsequently confirmed by a positive HIT antibody screening test. The patient’s platelet counts improved in a few days, and he was successfully transitioned to warfarin without complications. This case highlights the critical importance of maintaining a high index of suspicion for HIT in any patient exposed to Heparin products, including LMWH, who develops thrombocytopenia, even when the clinical presentation is atypical. Early recognition and prompt management with alternative anticoagulants are essential to prevent life-threatening thromboembolic complications.

## Introduction

Heparin-induced thrombocytopenia (HIT) is an uncommon but severe, immune-mediated adverse drug reaction characterized by the paradoxical combination of a falling platelet count and a prothrombotic state following heparin administration [1]. This condition arises from the formation of antibodies against complexes of platelet factor 4 (PF4) and heparin. These antibodies activate platelets via their FcγRIIA receptors, triggering a cascade of platelet aggregation, thrombin generation, and the release of procoagulant microparticles, which markedly increases the risk of life-threatening thromboembolic events such as deep vein thrombosis, pulmonary embolism, stroke, and limb ischemia [1].

The incidence of HIT varies depending on the type of heparin used and the clinical population. It is estimated to occur in 0.1% to 5% of patients receiving unfractionated heparin (UFH) but is considered less frequent with low-molecular-weight heparin (LMWH), with an estimated incidence of 0.1-1% [1]. This lower incidence rate with LMWH, while statistically significant, can inadvertently foster a clinical bias, leading to a decreased index of suspicion among clinicians when a patient on LMWH develops thrombocytopenia. This potential for diagnostic delay poses a serious risk, as the consequences of untreated HIT are equally severe regardless of the triggering heparin type.

This report details the case of a patient who developed HIT secondary to tinzaparin, an LMWH, following a recent vascular surgery. Tinzaparin is an anticoagulant that acts by inhibiting factor Xa and is commonly used for the prevention of post-operative venous thromboembolism and for the treatment of deep vein thrombosis and pulmonary embolism. The case is notable for its atypical presentation of syncope, which is not a classic sign of HIT, and it underscores the critical need for clinical vigilance. The purpose of this report is to emphasize that HIT should be considered in the differential diagnosis for any patient with thrombocytopenia and recent heparin exposure, highlighting the importance of early diagnosis and appropriate management to mitigate thrombotic risk [1].

## Case presentation

A 77-year-old male with a background of hypertension presented to the emergency department following an episode of syncope. He reported a preceding sensation of heaviness in both legs but denied chest pain, shortness of breath, or palpitations. His recent history was significant for an elective endovascular aneurysm repair (EVAR) performed a month earlier for an abdominal aortic aneurysm. A postoperative CT angiogram conducted 20 days after the procedure had revealed a thrombus in the left internal iliac graft limb, for which he was commenced on therapeutic tinzaparin (15,500 units once daily) as bridging therapy until a therapeutic international normalized ratio (INR) could be achieved on warfarin. Three days after starting tinzaparin, he developed the above symptoms and presented to the emergency department.

On initial examination, the patient was hemodynamically stable, and a systemic examination was unremarkable. Lying and standing blood pressure was measured, and orthostatic hypotension was excluded. A key challenge emerged early in the diagnostic process due to confusion regarding his anticoagulation status. The clinical team that assessed initially documented that he was only taking warfarin, which directed the initial diagnostic workup away from heparin-related complications. This illustrates a critical real-world challenge where incomplete medication reconciliation can delay the consideration of a correct diagnosis. Only after a repeat and detailed history-taking was it clarified that the patient was actively receiving tinzaparin injections and had not yet started warfarin.

**Figure 1:**
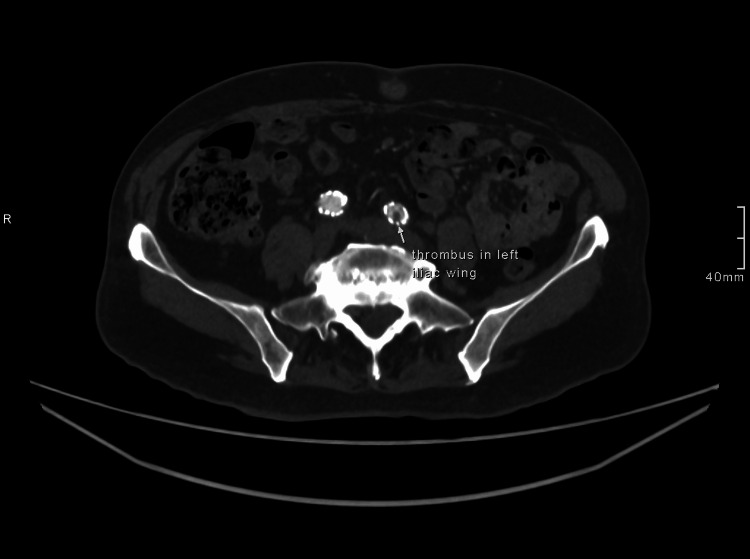
CT abdominal aorta with contrast showing thrombus in the left iliac wing of the graft

**Figure 2:**
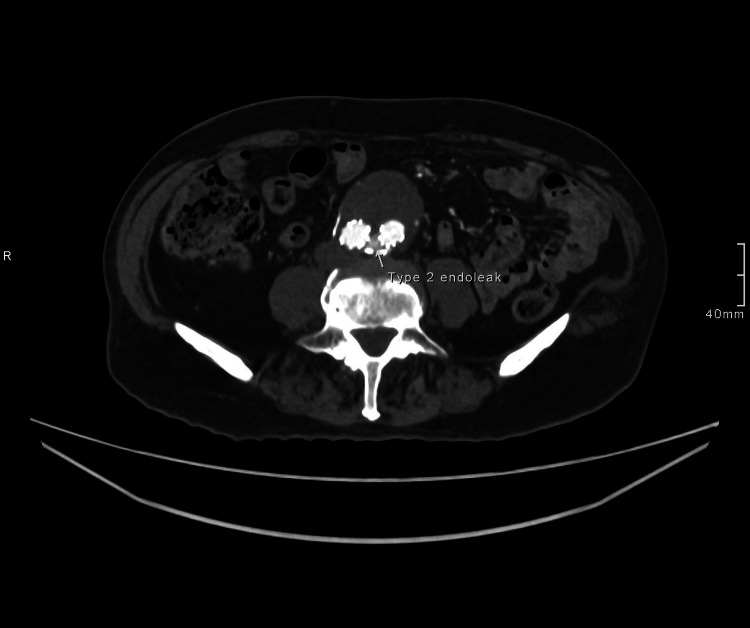
CT abdominal aorta with contrast showing type 2 endoleak

Initial investigations showed an unremarkable chest X-ray, with a normal electrocardiogram and 24-hour Holter monitoring, excluding cardiac causes of syncope. Laboratory results revealed isolated severe thrombocytopenia, with a platelet count of 44 × 10⁹/L. All other blood parameters, including liver and renal function tests as well as the bone profile, were within normal limits. A CT aortogram showed a small, slightly improved filling defect in the left iliac limb of the graft and a persistent small type II endoleak, as shown in Figures 1-2. The vascular surgery team considered these findings clinically insignificant.

**Figure 3:**
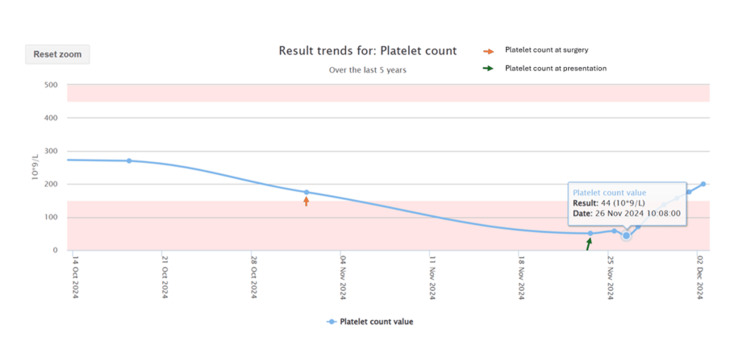
Trend of platelet count The count was 175 × 10⁹/L at the time of endovascular aneurysm repair (EVAR) surgery on the 31st of October but had fallen to 44 × 10⁹/L by the 26th of November, prompting discontinuation of tinzaparin. A gradual recovery was then observed, with platelet levels reaching 200 ×10⁹/L by the 2nd of December.

With the clarification of tinzaparin exposure, the development of thrombocytopenia three days after its initiation raised a high suspicion for HIT. The 4Ts score, a clinical prediction rule for HIT, was calculated to be 6 (thrombocytopenia: two points for a >50% fall; timing: two points for onset between days 5 and 10, but possible earlier with prior exposure; thrombosis: two points for new thrombosis; other causes: 0 points), indicating a high pre test probability of HIT. Based on this high suspicion, tinzaparin was immediately discontinued. The haematology team was consulted, and alternative anticoagulation with fondaparinux was initiated. A subsequent HIT antibody screening test returned positive with an HIT screen value of 9.86, confirming the diagnosis. The patient's clinical course was monitored with daily platelet counts, which showed a steady and progressive recovery, as detailed in Table 1 and Figure 3.

**Table 1:** Platelet count trend during hospitalization This table shows the daily platelet count from the day of admission, demonstrating a clear recovery trend after the cessation of tinzaparin on day 1 (26/11).

Date	Platelet count (10^9^/L)
26th November	44
27th November	71
28th November	110
29th November	137
30th November	157
1st December	176
2nd December	200

Once the platelet count recovered to over 50 x 10^9^/L, warfarin was initiated cautiously in conjunction with fondaparinux. The patient was closely monitored for any signs of warfarin-induced skin necrosis, a potential complication if warfarin is started prematurely in HIT, which did not occur. His platelet count normalized within six days of stopping tinzaparin. He remained hemodynamically stable throughout his admission and was discharged with a plan for outpatient six-month follow-up with the vascular surgery service. 

## Discussion

This case illustrates a clinically significant and atypical presentation of HIT induced by the LMWH tinzaparin. The report provides valuable insights into the diagnostic challenges, management strategies, and the overarching importance of maintaining a high index of suspicion for this rare but potentially life-threatening condition [1].

Significance of an atypical presentation

The patient's presenting symptom of syncope is not a classic feature of HIT, which more commonly manifests with signs of deep vein thrombosis, pulmonary embolism, or arterial thrombosis [1,2]. The syncope may have been related to a small, non-massive pulmonary embolus or a transient cardiac arrhythmia secondary to the hypercoagulable state induced by HIT. This atypical presentation underscores a crucial teaching point: HIT should be considered in any patient on heparin who presents with unexplained clinical deterioration accompanied by thrombocytopenia, even if the symptoms are not typical of thrombosis. Justifying the uniqueness of the case is a key component of a valuable case report, and the unusual presentation of syncope serves this purpose [2,3].

Pathogenesis and diagnosis in the context of LMWH

HIT is categorized into two types. Type I is a non-immune, mild, and transient thrombocytopenia that does not require cessation of heparin. By contrast, type II HIT, as seen in this patient, is an immune-mediated disorder with a high risk of thrombosis [1,4]. The pathogenesis involves IgG antibodies binding to PF4/heparin complexes, which then activate platelets and generate thrombin, creating a potent prothrombotic state [1,3]. Although this mechanism is identical for both unfractionated heparin (UFH) and LMWH, the clinical trigger in this case, tinzaparin, is less common, reinforcing the need to overcome the cognitive bias that LMWH is largely safe from this complication [5]. The 4Ts scoring system proved invaluable in this case, rapidly establishing a high pretest probability and guiding the crucial decision to stop tinzaparin immediately, even before laboratory confirmation was available [3,6]. This aligns with expert recommendations to use clinical scoring systems to guide initial management [3].

Management strategies and rationale for fondaparinux

The cornerstone of HIT management is the immediate cessation of all forms of heparin, including therapeutic and prophylactic doses, as well as heparin flushes and heparin-coated catheters [1,3]. This must be followed by the initiation of an alternative, non-heparin anticoagulant. Approved options include direct thrombin inhibitors (e.g., argatroban, bivalirudin) and the indirect factor Xa inhibitor fondaparinux [3,6]. The choice of fondaparinux in this case represents a common and practical therapeutic decision. Argatroban is cleared by the liver and requires continuous intravenous infusion with frequent monitoring, while fondaparinux is administered as a once-daily subcutaneous injection and is cleared by the kidneys [3,6]. In a patient with normal renal function, fondaparinux offers a simpler and effective management strategy.

The careful bridging to warfarin is another critical aspect of management. Starting warfarin alone in a patient with acute HIT can paradoxically worsen the prothrombotic state by depleting protein C before the vitamin K-dependent clotting factors, potentially leading to venous limb gangrene and skin necrosis [1,3,6]. Therefore, warfarin should only be initiated once the platelet count has shown substantial recovery (typically >150 × 10⁹/L, although >50 × 10⁹/L is sometimes used) and must overlap with the alternative anticoagulant for at least five days [3,7].

Literature context and limitations

This case adds to the body of evidence documenting HIT as a complication of LMWH. While large-scale adverse event reporting systems have logged thousands of suspected HIT cases, specific reports detailing Tinzaparin-induced HIT with an atypical presentation are less common. The case reinforces the incidence data suggesting that while the risk is lower with LMWH than with UFH, it is not zero [1,4,5].

The primary limitation of this report is that it is a single case study, and therefore, broad conclusions about the incidence or typical presentation of tinzaparin-induced HIT cannot be drawn. However, its value lies in its educational impact, serving as a potent reminder for clinicians to remain vigilant.

## Conclusions

This case of tinzaparin-induced thrombocytopenia HIT presenting as syncope underscores two critical clinical lessons. First, the index of suspicion for HIT must remain high for any patient on any form of heparin who develops thrombocytopenia, regardless of an atypical clinical presentation. The differential diagnosis should always include HIT in such scenarios, particularly in postoperative patients where multiple other causes of thrombocytopenia may exist. Second, the perceived lower risk of HIT with LMWH should not lead to diagnostic complacency. Prompt recognition, guided by clinical tools like the 4Ts score, followed by immediate cessation of the offending agent and initiation of a non-heparin anticoagulant such as fondaparinux, is paramount to preventing life-threatening thromboembolic events. This case reinforces the need for vigilance and a thorough understanding of this complex drug reaction to ensure patient safety in an increasingly complex medical environment.
